# Visual Rehabilitation in Combined Surgical Procedures: Bridging Two Eye Poles for Better Vision

**DOI:** 10.1155/2016/1265342

**Published:** 2016-08-18

**Authors:** M. J. Koss, C. Y. Choi, R. R. Krueger, M. Maia, H. B. Fam

**Affiliations:** ^1^Augenzentrum Nymphenburger Höfe and Augenklinik Herzog Carl Theodor, München, Germany; ^2^Department of Ophthalmology, University of Heidelberg, Heidelberg, Germany; ^3^Department of Ophthalmology, Kangbuk Samsung Hospital, Sungkyunkwan University School of Medicine, Seoul, Republic of Korea; ^4^Cleveland Clinic Foundation, Cole Eye Institute, Cleveland, OH, USA; ^5^Department of Ophthalmology, Universidade Federal de São Paulo, São Paulo, SP, Brazil; ^6^Department of Ophthalmology, Tan Tock Seng Hospital, Singapore

The starting idea of this special issue was to link innovative anterior to posterior ocular surgical procedures in an era of highly dynamic innovations in both segments of the eye. Every one of the editors had his own perception of what this might implicate and it turns out that all the submitting colleagues had scientific clinical and preclinical projects long before in place and were willing to share their outcomes with us. T. Tandogan et al., for example, illustrated to us the simple challenge of phacoemulsification after vitrectomy as a result of the altered intraocular fluid dynamics, a simple basic paper not only for the beginner, but also for every surgeon* before* he/she starts to perform combined procedures.

There is an intriguing argument for combined procedures—they abandon the need for a second operation! But this argument needs to be weighed with aspects like clinical feasibility, safety, and outcomes. The main promise, for instance, of 27-gauge vitrectomy is as follows: “the smaller you get, the lower is your wound, hence less postoperative complication rates.” Eagerly testing new techniques, each surgeon might then experience intraoperatively longer surgical maneuver times (depending on different manufacturers of course). We are thus glad about the manuscript on fluidics of the two-dimensional cutting (TDC) vitrectome by M. Pavlidis, who demonstrates to us how the cutter design makes all the difference for an effective 27-gauge vitrectomy.

All the contributing authors strived for better care by bridging clinical benchmarks like complication rates in minimal invasive small incision cataract and 23/25/27-gauge vitrectomies. F. Höhn et al. and R. M. Navarro et al. could nicely demonstrate both high safety measures and effective outcomes, in single-session phacovitrectomy procedures. The positive impact on visual acuity and quality of vision in patients with symptomatic floaters, who were implanted with a multifocal intraocular lens, will definitely be investigated by others with the introduction of newer multifocal IOLs in the future.

Others, like T. Bertelmann et al., could confirm higher safety of complicated cataract surgery in conjunction with retinal cryocoagulation in a rather large retrospective single-center clinical series. S. Deuchler et al. on the other hand could link complicated PVR retina surgeries with oil tamponades to the timing of a cataract surgery, while K. Nowomiejska et al. could demonstrate good retinal attachment with low corneal graft survival in severe trauma cases and combined vitrectomy, TKP, and corneal transplant surgery.

This special issue touches on many important clinical aspects of combined ocular procedures. We invite the designated readers to share our experiences with them.

